# *Anguillicola crassus* impairs the silvering-related enhancements of the ROS defense capacity in swimbladder tissue of the European eel (*Anguilla anguilla*)

**DOI:** 10.1007/s00360-016-0994-0

**Published:** 2016-05-04

**Authors:** Gabriel Schneebauer, Reinhold Hanel, Bernd Pelster

**Affiliations:** 1 Institut für Zoologie, Leopold-Franzens-Universität Innsbruck, Technikerstr. 25, 6020 Innsbruck, Austria; 2 Center for Molecular Biosciences, University of Innsbruck, Innsbruck, Austria; 3 Thünen Institute of Fisheries Ecology, Hamburg, Germany

**Keywords:** Reactive oxygen species, Swimbladder, European eel, Anguillicola crassus

## Abstract

In a process called silvering, European eels prepare for their long-distance migration from European freshwater systems to the Sargasso Sea for reproduction. During this journey, eels perform extended diel vertical migrations, and the concomitant changes in hydrostatic pressure significantly affect the swimbladder, functioning as a buoyancy organ. As the swimbladder is primarily filled with oxygen, the tissue has to cope with extreme hyperoxic conditions, which typically are accompanied by the generation of reactive oxygen species (ROS) and oxidative stress. In addition, since the introduction of the parasitic nematode *Anguillicola crassus* in the early 1980s, swimbladder function of most of the European eels is impaired by the infection with this parasite. However, the exact pathways to detoxify ROS and how these pathways are affected by silvering or the infection are still unknown. In swimbladder and muscle tissue from uninfected and infected yellow, and from uninfected and infected silver eels, we measured the level of lipid peroxidation, which increases with ROS stress. To assess the capacity of the ROS defense systems, we analyzed the activities of superoxide dismutase (SOD), catalase, glutathione peroxidase (GPx) and glutathione reductase (GR), and determined the concentration of the antioxidant glutathione (GSH + GSSG). In swimbladder tissue, we found increased concentrations of GSH + GSSG as well as higher activities of SOD, GPx and GR, suggesting that SOD and the glutathione cycle are important for ROS detoxification. Comparing swimbladder tissue of uninfected yellow with uninfected silver eels, the concentration of GSH + GSSG and the activity of SOD were higher after silvering, corresponding with lower levels of lipid peroxidation. Whereas in yellow eels the infection with *A. crassus* had no effect, in silver eels the capacity to cope with ROS was significantly impaired. In muscle tissue, silvering or the infection only affected the activity of SOD but in exactly the same way as in swimbladder tissue.

**Electronic supplementary material:**

The online version of this article (doi:10.1007/s00360-016-0994-0) contains supplementary material, which is available to authorized users.

## Introduction

Since the 1980s, recruitment of European eels has decreased by about 95 % (ICES 2015). As a consequence, *A.* *anguilla* is listed as critically endangered species by the International Union for the Conservation of Nature and Natural Resources since 2010 (Jacoby and Gollock 2014). Due to its complex lifecycle, this species is exposed to a wide range of potential stressors which almost certain act synergistically (Wirth and Bernatchez 2003), such as changes in ocean currents (Baltazar-Soares et al. 2014), increasing sea surface temperature with a concomitant decline in primary production (Bonhommeau et al. 2008), pollution (Geeraerts and Belpaire 2010), habitat loss (Kettle et al. 2011), overfishing (Dekker 2004), and parasitism (Lefebvre et al. 2012; Bandín et al. 2014).

The complex life cycle of the European eel (*Anguilla anguilla*) actually starts in the Sargasso Sea, although the catadromous fish spends most of its lifetime in European freshwater systems as so called yellow eel. After several years of growth, eels return to the coast and in a process called silvering, prepare for their long-distance migration back to the Sargasso Sea, the expected spawning ground of this species (Schmidt 1923; Miller et al. 2015). Silvering includes, for example, changes in body color, enlargement of the eyes with a concomitant increase in the number of rods on the retina, appearance of neuromasts along the lateral line, increase of body fat content, and adjustment of ionoregulatory and osmoregulatory processes for a life in seawater (Tesch et al. 2003; van Ginneken and Maes 2005; Righton et al. 2012).

During their spawning migration, eels perform diel vertical migrations, swimming at depths of 600–1000 m during daytime and depths of 100–300 m at night (Aarestrup et al. 2009; Wysujack et al. 2015). Accordingly, migrating eels are exposed to various hydrostatic pressures of up to 101 atm. The effects of short and long-term exposure to high hydrostatic pressure on energy metabolism, membrane properties, and muscle tissue of eels have been analyzed repeatedly (Sébert et al. 1991; Scaion et al. 2008; Sébert et al. 2009b). The results revealed that oxygen consumption decreases after long-term exposure to a pressure of 101 atm, and swimming efficiency appears to be improved under elevated pressure (Sébert et al. 2009a). The effect of elevated hydrostatic pressure on swimbladder tissue has not yet been addressed.

The gas-filled swimbladder is functioning as a buoyancy organ (Fänge 1983; Pelster 1997, 2013). Accordingly, pressure and volume of the swimbladder are significantly affected by the changes in hydrostatic pressure encountered during vertical migrations (Pelster 2015). To provide neutral buoyancy, the swimbladder volume should be kept constant in spite of the changing hydrostatic pressure. Silvering has indeed been shown to improve swimbladder function by increasing wall thickness and vascularization, deposition of guanine into the swimbladder wall to reduce diffusional gas loss and enlarging the *retia mirabilia* to improve countercurrent concentration ability (Kleckner 1980a, b; Yamada 2001). In the American eel (*Anguilla rostrata*) these improvements, for example, lead to a fivefold increase in gas deposition rate (Kleckner 1980a).

The swimbladder of the European eel is primarily filled with oxygen (Kobayashi et al. 1990), and as eels reach depths of up to 1000 m during their diel vertical migrations on their journey to the spawning grounds in the Sargasso Sea (Aarestrup et al. 2009; Wysujack et al. 2015), we expect extreme hyperoxic conditions inside the swimbladder and in gas gland tissue (Pelster 1997, 2011, 2015). Hyperoxic conditions, however, stimulate the generation of reactive oxygen species (ROS) (Lushchak and Bagnyukova 2006) and have been shown to induce oxidative stress, for example, in goldfish *Carassius auratus* (Lushchak et al. 2005a), Atlantic salmon *Salmo salar* (Olsvik et al. 2005) and Senegal sole *Solea senegalensis* (Salas-Leiton et al. 2009). Reactive oxygen species are primarily generated at the inner mitochondrial membrane when electrons escape the respiratory chain (Balaban et al. 2005). They interact with molecular oxygen and form the superoxide anion radical (O_2_˙^−^), which is then converted into hydrogen peroxide (H_2_O_2_) and thereafter to hydroxyl anion (OH˙) (Valko et al. 2007; Lushchak 2014). ROS can be involved in several cellular signaling pathways including cell cycle, stress response or energy metabolism, but when not controlled by antioxidants, the developing oxidative stress can result in lipid peroxidation, protein carbonylation and modifications of nucleic acids (Dröge 2002; Lushchak 2011). Therefore, an unregulated accumulation of ROS in cells can result in massive tissue damage.

Oxidative stress is not solely induced by hyperoxia, but also by much more common hypoxia-reoxygenation events, and animals have developed elaborate mechanisms to detoxify ROS (Welker et al. 2013; Lushchak 2014). Non-enzymatic antioxidants include ascorbic acid (vitamin C), α-tocopherol (vitamin E), glutathione (GSH), carotenoids or flavonoids, whereas enzymatic antioxidants involve superoxide dismutase (SOD), glutathione peroxidase (GPx) and catalase (Cat). For detoxification SOD converts two molecules O_2_˙^−^ to O_2_ and H_2_O_2_ which is then either broken down to H_2_O and O_2_ by Cat or reduced to H_2_O by GPx, using GSH. Utilizing NADPH, oxidized glutathione (GSSG) in turn is reduced to GSH by glutathione reductase (GR) to maintain the pool of GSH (Dröge 2002; Valko et al. 2007; Lushchak 2014).

A nematode significantly impairing swimbladder function is *Anguillicola crassus*. The parasitic nematode was introduced to Germany with the importation of infected Japanese eels (*Anguillajaponica*) from Taiwan in 1980 and has spread all over Europe within 10 years, infecting most of the European eels (Kirk 2003; Lefebvre et al. 2013). *A. crassus* invades the swimbladder and affects its function by impairing gas deposition, feeding on swimbladder tissue, causing wall thickening, inflammation, tissue degeneration, and filling the lumen with eggs, larvae and dead nematodes. Ultimately, these effects can lead to a total loss of function (Würtz et al. 1996; Würtz and Taraschewski 2000; Kirk 2003; Kennedy 2007; Barry et al. 2014).

Based on these considerations we hypothesized that swimbladder tissue of the European eel would be equipped with an advanced ROS detoxification system compared with muscle tissue and that silvering would improve, whereas an infection with *A. crassus* would impair, this defense system. White muscle tissue was used for comparison because this tissue typically is not very well perfused and therefore not exposed to elevated oxygen pressure and not particularly prone to ROS production as shown in goldfish *Carassius auratus* and common carp *Cyprinus carpio* (Lushchak et al. 2005a, b).

To test these hypotheses, we examined swimbladder and muscle tissue of uninfected and infected yellow eels, and compared it with uninfected and infected silver eels caught in the transition zone between fresh- and seawater for various components of the ROS detoxification system and for an oxidative stress marker. Although these silver eels are not yet exposed to the high hydrostatic pressures encountered in the open ocean, they are preparing for this migration and therefore are expected to show an improved ROS defense in the swimbladder. We found that the ROS defense capacity was much higher in swimbladder tissue compared with muscle tissue and that silvering and an infection with *A. crassus* significantly affect this capacity.

## Materials and methods

### Animals

All experiments were performed with European eels (*A. anguilla*). 48 yellow eels were caught in May by local fisherman using fish bottom traps in Lake Constance, Bregenz, Austria, and kept in an outdoor freshwater basin at the Institute of Zoology at the University Innsbruck, not exceeding 3 weeks. Two days prior to sampling, fish were transferred into an indoor freshwater aquarium. Mean body mass of uninfected and infected yellow eels, chosen for the experiments, was 470 ± 213 g (*N* = 6) and 510 ± 200 g (*N* = 6), respectively. 30 silver eels were caught by local fishermen in the open water of the River Elbe, close to Winsen (Luhe), Germany, and 15 in the Baltic Sea, off the coast of Fehmarn, Germany, and kept in an outdoor basin with a freshwater supply at the Thünen Institute of Fisheries Ecology, Ahrensburg, Germany, until sampling, not exceeding 7 days. Table 1 shows the morphometrics of uninfected and infected silver eels chosen for the experiments. The silver index was calculated according to Durif et al. (2005), and the eye index according to Pankhurst (1982). There was no difference in the silver index between silver eels from the two catching locations. In yellow eels obtained from Lake Constance the eyes were small, differentiated neuromasts were not visible and the high color contrast, which would be typical for silver eels, was not given. Based on these observations these fish were classified as yellow eels (Acou et al. 2005).

**Table 1:** Morphometrics, silvering index calculated according to Durif et al. (2005) and ocular index calculated according to Pankhurst (1982) of uninfected and infected silver eels

	Silver uninfected	Silver infected
Body mass (kg)	1.078 ± 0.306	0.889 ± 0.332
Body length (cm)	80.86 ± 4.63	77.33 ± 7.89
Pectoral fin length (mm)	37.69 ± 1.60	37.78 ± 6.02
Horizontal eye diameter (mm)	9.79 ± 0.83	9.82 ± 0.73
Vertical eye diameter (mm)	9.21 ± 0.25	9.30 ± 0.77
Silver index	4.14 ± 0.69	4.00 ± 0.89
Ocular index	8.77 ± 0.70	9.35 ± 1.10

Overall mean values ± SD; *N* = 6

### Tissue preparation

Eels were anesthetized with neutralized tricaine methanesulfonate (MS-222) on ice and subsequently decerebrated and spinally pithed. The abdominal wall was opened ventrally, the swimbladder carefully dissected and cleaned from connective tissue. After removal, the remaining swimbladder epithelium was rinsed, cleaned from blood, debris and nematodes if necessary. Rinsed and afterwards quickly dried on absorbent paper, the tissue was immediately shock frozen in liquid nitrogen. Swimbladder tissue preparation and cleaning took no more than 2–3 min. White muscle tissue samples were obtained from the abdominal wall close to the retia mirabilia and immediately shock frozen in liquid nitrogen. Tissues were then stored at −80 °C until further use. After completing tissue preparation, the number of nematodes isolated from infected swimbladders was counted. Yellow eels with no (*N* = 5) or 1 small (length about 4 mm) *A. crassus* (*N* = 1) inside the swimbladder without visible modifications of the swimbladder wall were considered as uninfected (N = 6 in total). In these eels, after peeling off the connective tissue the swimbladder wall was transparent and thin. Eels with eight or more *A. crassus* inside the swimbladder were counted as infected. In the yellow eel group were one animal with 10 nematodes, three animals with 12 nematodes, one animal with 24 nematodes, and one animal with 30 nematodes in the swimbladder. In infected silver eels we counted between 8 and 77 nematodes (2 × 8, 1 × 9, 1 × 25, 1 × 36, 1 × 77). In these fish, the swimbladder contained exudate, was comparatively small, thick walled and had an opaque appearance. For each group, uninfected yellow, infected yellow, uninfected silver and infected silver, muscle and swimbladder tissue of six animals were analyzed (*N* = 6). Tissue sampling was performed in compliance with the Austrian law and the guidelines of the Austrian Federal Minister for Education, Arts and Culture.

### Biochemical analysis

For determination of total glutathione (GSH + GSSG) content, 50 mg swimbladder or muscle tissue were homogenized in 500 µl 5 % metaphosphoric acid (MPA) in Precellys tubes CKMix (No. KT03961-1-009.2), with a Precellys 24 homogenizer (Bertin Technologies, France) (30 s at 5900 rpm). The obtained homogenates were centrifuged at 12,000*g* for 15 min at 4 °C and the supernatants stored at −80 °C until further use. For GSH + GSSG determination the OxiSelect total glutathione (GSSG/GSH) Assay Kit (STA-312) (Cell Biolabs, Inc., San Diego, USA) was used, following the manufacturer’s instructions. Appropriate dilutions of the supernatants were analyzed using a VICTOR™ X4 Multilabel Plate Reader (PerkinElmer, Inc., Waltham, MA, USA).

For determination of enzyme activities, 75 mg swimbladder or muscle tissue were homogenized in 500 µl lysis buffer (10 mM Tris, 150 mM NaCl, 0.1 mM EDTA, pH 7.5) in Precellys tubes CKMix with a Precellys 24 homogenizer (30 s at 5900 rpm). The obtained homogenates were centrifuged at 12,000*g* for 10 min at 4 °C and the supernatants stored at −80 °C until further use. For measuring glutathione peroxidase (GPx; EC 1.11.1.9.), glutathione reductase (GR; EC 1.6.4.2.), catalase (Cat; EC 1.11.1.6.) and superoxide dismutase (SOD; EC 1.15.1.1.) the Glutathione Peroxidase Assay Kit (No. 703102; Cayman Chemical Company, Ann Harbor, MI, USA), the Glutathione Reductase Assay Kit (No. 703202; Cayman Chemical Company, Ann Harbor, MI, USA), the Amplex Red Catalase Assay Kit (A22180; Molecular Probes, Eugene, OR, USA) and the SOD Determination Kit (No. 19160; Sigma-Aldrich Co. LLC., St. Louis, MO, USA) were used, respectively, following the manufacturer’s instructions. Appropriate dilutions of the supernatants were analyzed using a VICTOR™ X4 Multilabel Plate Reader. One unit of GPx, GR and Cat activity was defined as the amount of the enzyme generating 1 µmol of product or consuming 1 µmol of substrate per minute. One unit of SOD was defined as the amount of the enzyme causing a 50 % inhibition of a superoxide anion coupled colorimetric reaction. Activities were expressed as units (or milliunits) per milligram protein.

For determination of malondialdehyde (MDA), 100 mg swimbladder or muscle tissue, respectively, were homogenized in 1 ml butylated hydroxytoluene in PBS (according to the manual) in Precellys tubes CKMix with a Precellys 24 homogenizer (2 × 30 s at 5900 rpm with 120 s break). The obtained homogenates were centrifuged at 10,000*g* for 5 min at 4 °C and the supernatants stored at −80 °C until further use. MDA is a low-molecular weight end product formed via the decomposition of lipid peroxidation products and therefore used to assess the rate of lipid peroxidation (Janero 1990). Appropriate dilutions of the supernatant were measured using the OxiSelect™ TBARS Assay Kit (STA-330) (Cell Biolabs, Inc., San Diego, USA) and a VICTOR™ X4 Multilabel Plate Reader.

Total protein concentration in all but the MPA homogenates was determined using a Nanodrop 2000c Spectrophotometer (Thermo Scientific, Waltham, MA, USA).

### Statistics

Data are presented as mean ± SD with *N* giving the number of animals analyzed. GSH + GSSG concentrations are given as nmol g^−1^ wwt (wet weight), MDA concentrations as nmol mg^−1^ protein and enzymes activities as U mg^−1^ protein. Shapiro–Wilk tests were performed using IBM SPSS Statistics Version 21.0 (IBM Corp., Armonk, NY, USA), to test data of experimental groups for normal distribution. As some groups failed the normality test, non-parametric Mann–Whitney *U*, Wilcoxon signed rank, and Spearman’s correlation tests were performed in SPSS to detect significant differences between independent groups, between related groups, and correlations, respectively. Redundancy analyses (RDA), which represent principal component analyses (Ter Braak and Šmilauer 2012), were performed using the CANOCO 5 for windows software package (Microcomputer Power, Ithaca, NY, USA). Significance of differences was accepted when *P* < 0.05.

## Results

**Fig. 1:**
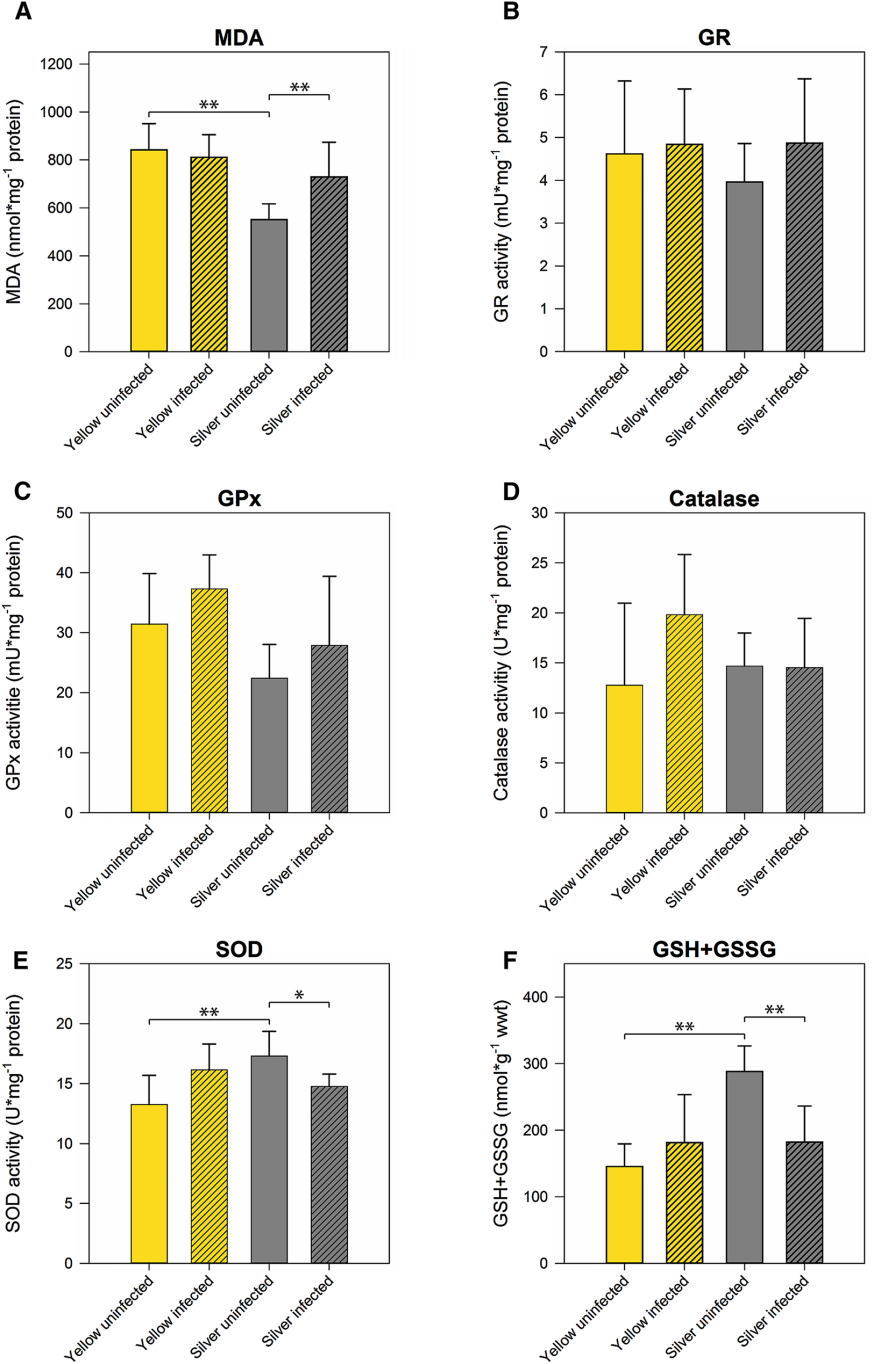
Activity of ROS-related enzymes and metabolite concentrations in swimbladder tissue of different development and infection status. **a** malondialdehyde, **b** glutathione reductase, **c** glutathione peroxidase, **d** catalase, **e** superoxide dismutase, **f** total glutathione (GSH + GSSG). *Bars* represent mean values ± SD (*N* = 6). *P < 0.05, **P < 0.01

To assess the level of oxidative stress in muscle and swimbladder tissue in yellow and silver eel, we measured MDA as an indicator of lipid peroxidation. In muscle tissue, MDA level was below the detection threshold, while in swimbladder tissue MDA was detectable (Fig. 1a). In uninfected silver eel swimbladder tissue, MDA concentration was significantly lower than in uninfected yellow eel swimbladder with 550.96 ± 65.39 nmol mg^−1^ protein and 842.36 ± 108.75 nmol mg^−1^ protein, respectively. In infected silver eel swimbladder, the MDA concentration was significantly higher than in uninfected silver eel, and no difference was detected between uninfected and infected yellow eel swimbladder, and infected eel swimbladder.

**Fig. 2:**
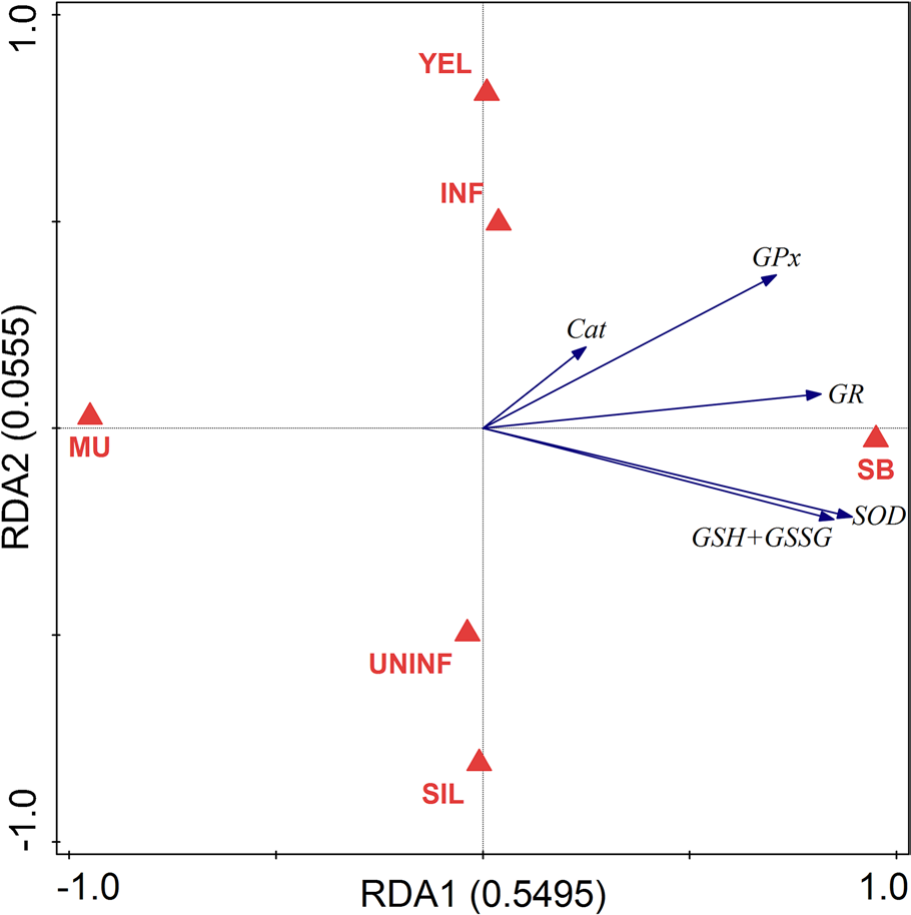
Redundancy analysis containing all explanatory and response variables. Eigenvalues (explained variation) given with axis labels. *YEL* yellow eel, *SIL* silver eel, *MU* muscle, *SB* swimbladder, *INF* infected, *UNINF* not infected. Significances of effects determined by CANOCO Monte-Carlo tests (4999 permutations). Pseudo-*F* = 23.2, *P* = 0.0002

To evaluate the antioxidative capacity of swimbladder tissue, we measured total glutathione concentration (GSH + GSSG), as well as GR, GPx, SOD, and Cat activity in swimbladder tissue and compared it with muscle tissue which is typically not exposed to high oxygen partial pressures. Disregarding developmental stage or infection status within both tissues, we found significantly higher concentrations of GSH + GSSG (+605 %) as well as higher activities of GR (+185 %), GPx (+148 %), and SOD (+159 %) in swimbladder tissue compared with muscle tissue whereas Cat activity was not significantly different between the tested tissues (Table 2). These differences between muscle and swimbladder tissue were detected within each of the four tested groups of eels as demonstrated by the results of paired *t* tests (Suppl. Fig. 1). RDA with tissue, developmental stage and infection status as explanatory variables and activities of GR, GPx, Cat, and SOD as well as GSH + GSSG concentration as response variables showed that 55 % of the overall variation could be attributed to the tissue, and only 6 % to the developmental stage or the infection status (Fig. 2). Using Spearman’s correlation test no correlation between the number of nematodes found in the swimbladder and any of the tested parameters was detected (*P* > 0.05), except for GSH + GSSG in infected eels with *R* = −0.582, *P* = 0.047. Yellow eels on average had a lower body mass than silver eels, but only for GR within silver eels a correlation between activity and body mass was detected (*R* = −0.683; *P* = 0.014). Within silver eels no correlation was detected between silver index and any of the tested parameters.

**Table 2:** Concentrations of metabolites and activities of various enzymes involved in ROS detoxification

	Muscle tissue	Swimbladder tissue
GSH + GSSG (nmol g^−1^ wwt)	28.26 ± 3.51	199.36 ± 61.60*
GR (mU mg^−1^ protein)	1.60 ± 0.27	4.57 ± 0.43*
GPx (mU mg^−1^ protein)	12.01 ± 5.42	29.76 ± 6,25*
SOD (U mg^−1^ protein)	5.93 ± 2.12	15.37 ± 1.75*
Catalase (U mg^−1^ protein)	12.56 ± 1.76	15.44 ± 3.05

Overall mean values ± SD; *N* = 24

* Significant differences between tissues (*P* < 0.001)

**Fig. 3:**
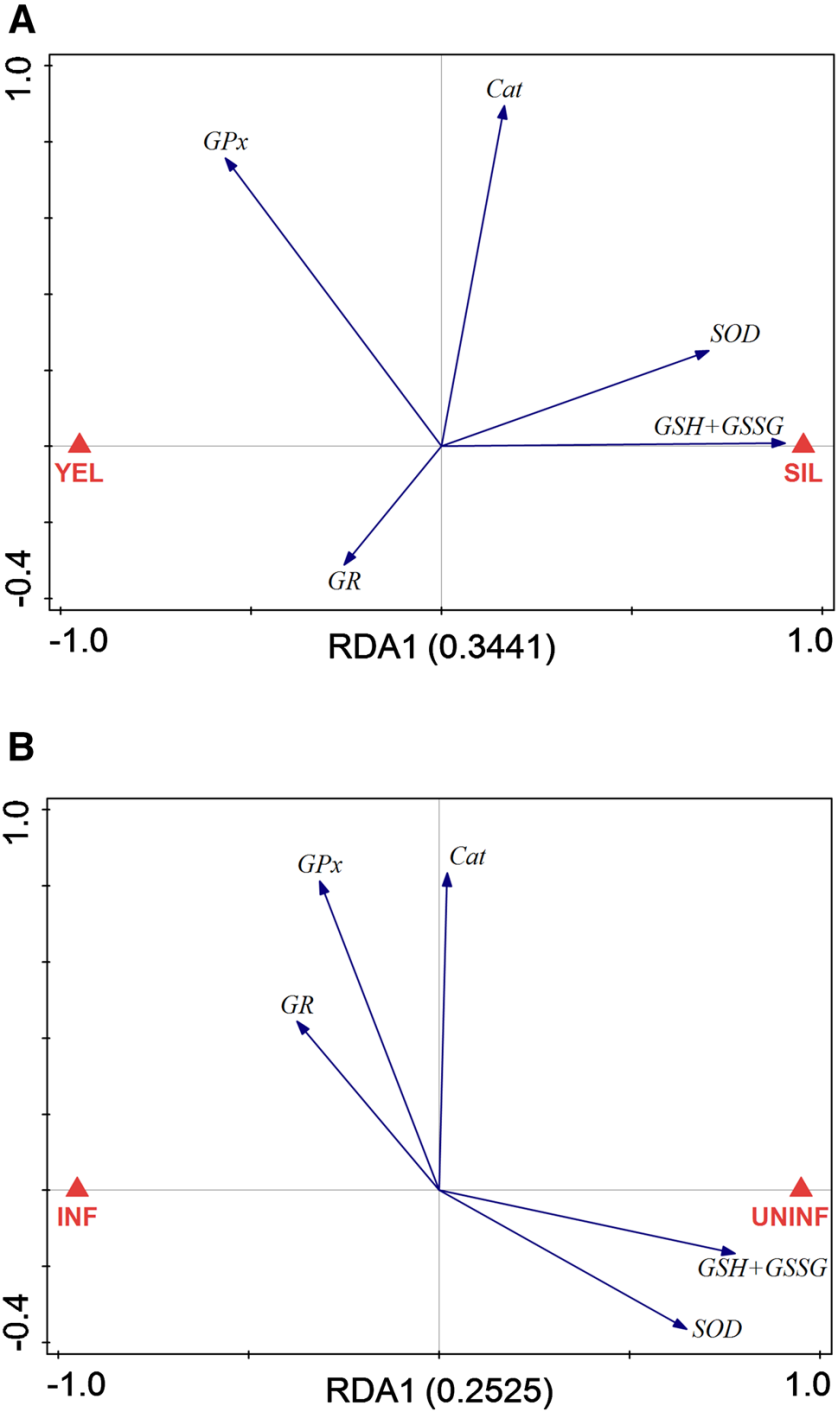
Redundancy analyses containing developmental stage within uninfected eels (**a**) or infection status within silver eels (**b**) as explanatory variable and all response variables in swimbladder tissue. Eigenvalues (explained variation) given with axis labels. *YEL* yellow eel, *SIL* silver eel, *INF* infected, *UNINF* not infected. Significances of effects determined by CANOCO Monte-Carlo tests (4999 permutations). Pseudo-*F* = 5.2, *P* = 0.0022 (**a**), Pseudo-*F* = 3.4, *P* = 0.0296 (**b**)

To assess the effect of silvering on the antioxidative capacity of swimbladder tissue, we compared total glutathione concentration (GSH + GSSG), as well as GR, GPx, SOD and Cat activity between uninfected yellow eels and uninfected silver eels. In swimbladder tissue, GR, GPx, and Cat activity were not significantly different between the tested developmental stages (Fig. 1b–d). In contrast, SOD activity in uninfected silver eels with 17.30 ± 2.05 U mg^−1^ protein was significantly higher compared with 13.26 ± 2.43 U mg^−1^ protein in uninfected yellow eels (Fig. 1e). The concentration of GSH + GSSG was also significantly higher in uninfected silver eels with 288.11 ± 38.43 nmol g^−1^ wwt compared with 145.52 ± 34.03 nmol g^−1^ wwt in uninfected yellow eels (Fig. 1f). RDA of uninfected swimbladder tissue showed that 34 % of the variation could be attributed to the developmental stage with SOD activity and GSH + GSSG concentration showing the strongest reaction with respect to silvering (Fig. 3a).

Focusing on the effect of an infection on the antioxidative capacity of swimbladder tissue, revealed that none of the measured enzyme activities or metabolite concentrations showed any significant difference within yellow eels (Fig. 1b–f). On the contrary, in infected silver eels the activity of SOD with 14.77 ± 1.03 U mg^−1^ protein was significantly decreased compared with 17.30 ± 2.05 U mg^−1^ protein in uninfected silver eels with (Fig. 1e). In addition, the concentration of GSH + GSSG was lower in infected silver eels with 182.30 ± 53.90 nmol g^−1^ wwt compared with 288.11 ± 38.43 nmol g^−1^ wwt in uninfected silver eels (Fig. 1f). Using RDA to assess the effect of the infection of the swimbladder with nematodes in silver eel showed that 25 % of the variation could be attributed to the infection status, and again SOD activity and GSH + GSSG concentration showed the strongest reaction (Fig. 3b).

**Fig. 4:**
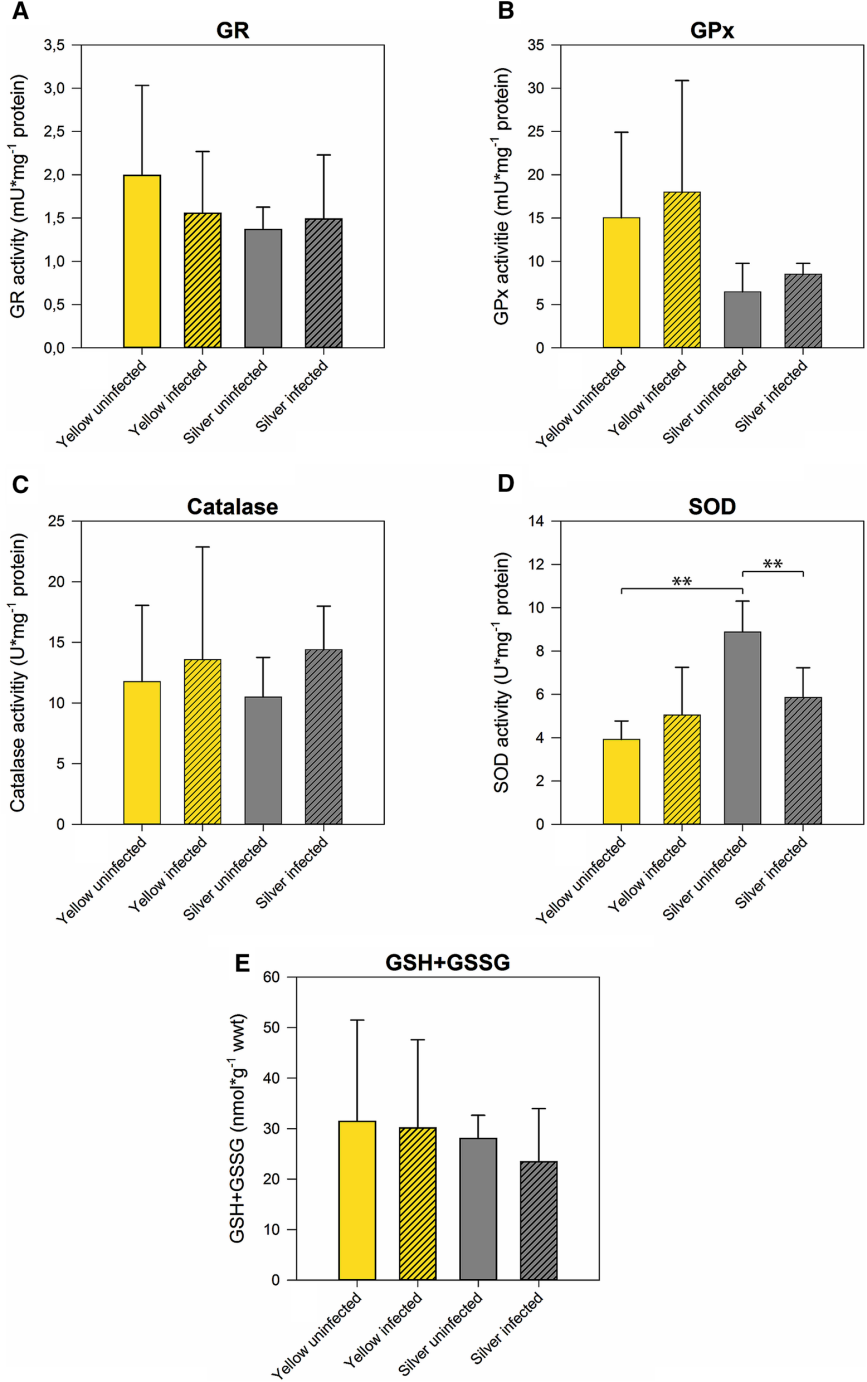
Activity of ROS-related enzymes and metabolite concentrations in muscle tissue of different development and infection status. **a** glutathione reductase, **b** glutathione peroxidase, **c** catalase, **d** superoxide dismutase, **e** total glutathione (GSH + GSSG). *Bars* represent mean values ± SD (*N* = 6). **P < 0.01

**Fig. 5:**
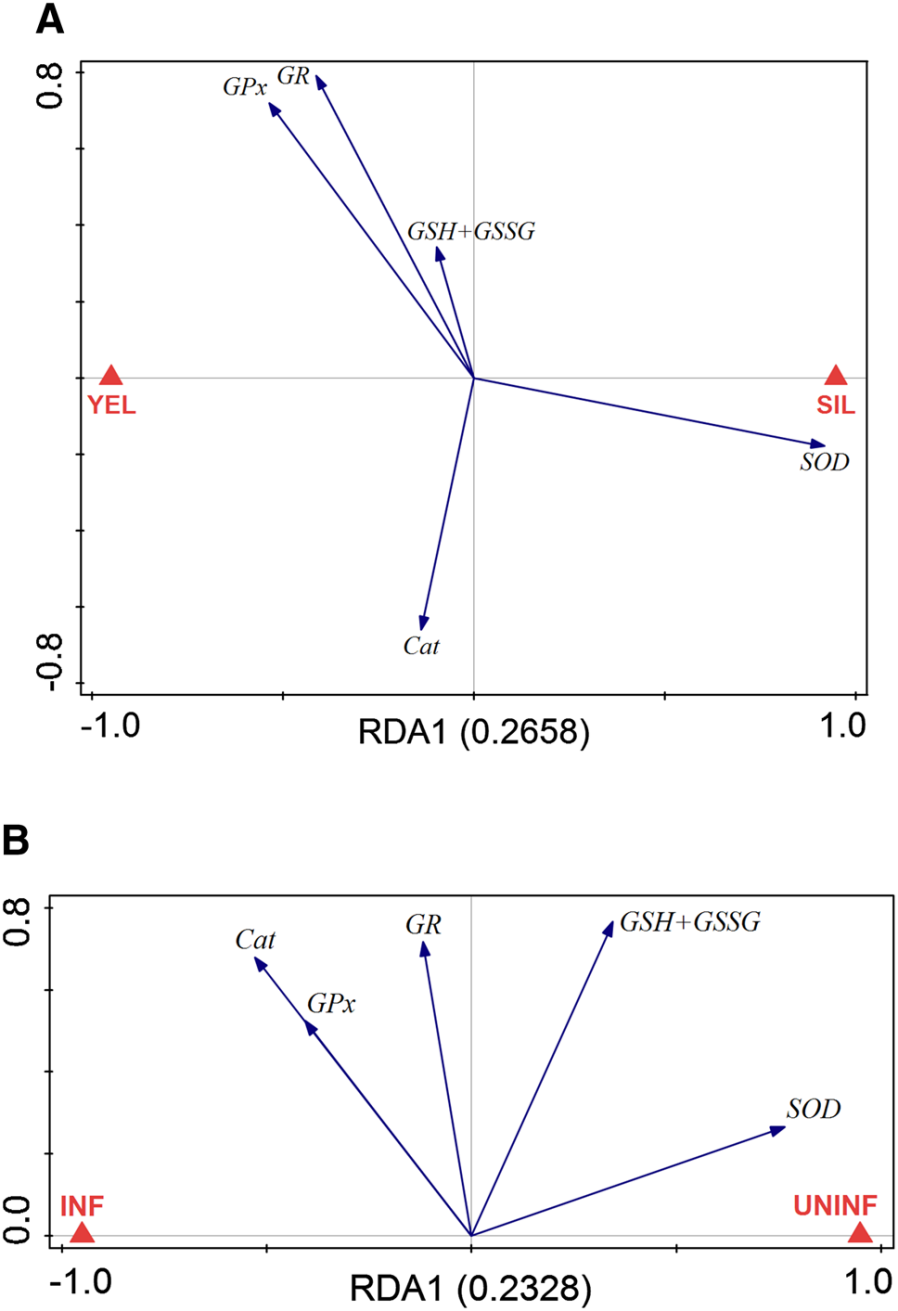
Redundancy analyses containing developmental stage within uninfected eels (**a**) or infection status within silver eels (**b**) as explanatory variable and all response variables in muscle tissue. Eigenvalues (explained variation) given with axis labels. *YEL* yellow eel, *SIL* silver eel, *INF* infected, *UNINF* not infected. Significances of effects determined by CANOCO Monte-Carlo tests (4999 permutations). Pseudo-*F* = 3.6, *P* = 0.0022 (**a**), Pseudo-*F* = 3.0, *P* = 0.0242 (**b**)

In muscle tissue, GR, GPx, and Cat activity, and the concentration of GSH + GSSG were not significantly different between the tested developmental stages (Fig. 4a–c, e). SOD activity, however, in uninfected silver eel muscle with 8.88 ± 1.42 U mg^−1^ protein was significantly higher compared with uninfected yellow eels with 3.92 ± 0.86 U mg^−1^ protein (Fig. 4d). Looking at the effect of the nematode infection on GR, GPx, and Cat activity and on GSH + GSSG concentration revealed no difference in yellow and in silver eel. SOD activity, however, was significantly lower in infected silver eel muscle and no longer different from the activity measured in uninfected or infected yellow eel muscle. RDA analysis demonstrated that 27 % of the variation could be attributed to the developmental stage, and SOD activity showed the strongest reaction (Fig. 5a), while 23 % of the variation could attributed to the effect of the infection on muscle tissue, and again SOD activity showed the strongest reaction (Fig. 5b).

## Discussion

In addition to the already described deleterious effects of an infection with *A. crassus* (Würtz et al. 1996; Würtz and Taraschewski 2000; Kirk 2003; Kennedy 2007; Barry et al. 2014), the significant impairment of the ROS defense capacity that we observed in our study underlines the potential contribution of this parasitic nematode to the decline of the European eel population.

The swimbladder has been shown to be mainly filled with oxygen (Kobayashi et al. 1990), so that compared with other tissues swimbladder tissue is exposed to much higher oxygen partial pressures. Exposure of tissues to high oxygen partial pressures, in turn, has been shown to result in higher levels of ROS production (Welker et al. 2013; Lushchak 2014). This assumption was confirmed by the assessment of lipid peroxidation using the MDA reaction. While in muscle tissue, MDA levels were below the detection limit of the used method, in swimbladder tissue low levels of lipid peroxidation were detected. In consequence, a better ROS defense capacity was expected for swimbladder tissue compared with muscle tissue. The significantly higher activities of SOD, GR and GPx, as well as the higher concentration of GSH + GSSG in swimbladder tissue as compared with muscle tissue supported this hypothesis and suggested SOD and the glutathione cycle to be of particular importance for ROS detoxification. Catalase activity, decomposing 2H_2_O_2_ to O_2_ and H_2_O, showed no significant difference between the tissues analyzed, suggesting that activity of this enzyme is not elevated in response to elevated oxygen tensions in swimbladder tissue. Nevertheless, our measured Cat activities for swimbladder tissue were in line with results of the physoclistous fish *Opsanus tau* and the American eel (*Anguilla rostrata*) (Morris and Albright 1981, 1984), suggesting that Cat activity found in yellow eel swimbladder tissue is sufficient also for the silver eel. Based on the significantly higher activities of GR and GPx, as well as the higher concentration of GSH + GSSG in swimbladder tissue, it can be concluded that GPx activity, the second peroxide metabolizing enzyme, and glutathione oxidation/reduction are more important in swimbladder tissue for the break-down of H_2_O_2_ than Cat activity. In goldfish *Carassius auratus* brain, liver and muscle tissue, GR activity significantly increased during recovery from hyperoxia, and the same was true for Glutathione-S-transferase in liver, kidney and brain and for GPx in in liver and kidney (Lushchak et al. 2005a). The GPx reaction could be the preferential route for detoxification of H_2_O_2_ as it is widely distributed in the cytosol, has a higher affinity for H_2_O_2_ than Cat and is also involved in the metabolization of lipid peroxides (Izawa et al. 1996; Lushchak et al. 2005a). A potential disadvantage of the glutathione system is the requirement for NADPH to reduce oxidized glutathione (GSSG) to GSH by glutathione reductase and the concomitant accumulation of NADP^+^. However, in gas gland cells of the toadfish *Opsanus beta* and of the European eel a high activity of the pentose phosphate shunt has been found (Walsh and Milligan 1993; Pelster et al. 1994). The pentose phosphate shunt generates CO_2_, which is used for gas deposition, but it also results in the reduction of NADP^+^. NADPH in turn can be used by GR for the conversion of GSSG to reduced GSH, thus maintaining the GSH pool.

The process of silvering improves swimbladder function in various ways, for example by increasing wall thickness and vascularization or by enlarging the *retia mirabilia* to improve countercurrent concentration ability (Kleckner 1980a, b; Yamada 2001). In our study we could show that ROS detoxification capacity is also enhanced, as demonstrated by an increased activity of SOD, which converts O_2_˙^−^ to O_2_ and H_2_O_2_, and the higher concentrations of GSH + GSSG, involved in the break-down of H_2_O_2_ to H_2_O and O_2_. Neither the activity of GR, nor of GPx were elevated in silver eel swimbladder tissue, suggesting that the activity established in yellow eel swimbladder tissue is sufficient to cope with the amount of ROS present in silver eel swimbladder before experiencing the very high oxygen partial pressures encountered during the diurnal vertical migrations of the spawning migrations (Aarestrup et al. 2009; Wysujack et al. 2015). The concurrent decrease in lipid peroxidation detected on the basis of the MDA assay represents a first indication for decreased oxidative stress due to the enhanced ROS defense. Catalase again appears not to play a prominent role in ROS defense in silver eel swimbladder tissue as a significant change of activity could not be observed.

Severe infections of the eel swimbladder with the parasitic nematode *A. crassus* have been shown to clearly damage tissue and to impair swimbladder function (Würtz et al. 1996; Würtz and Taraschewski 2000; Kirk 2003; Kennedy 2007; Barry et al. 2014). Our present results show that an infection also negatively affected ROS detoxification in the silver eel swimbladder. SOD activity was significantly decreased in infected silver eel swimbladder tissue as compared with uninfected silver eels, and also the concentration of GSH + GSSG was significantly lower. Hence, the detoxification capacity for both reactive oxygen species, O_2_˙^−^ and H_2_O_2_ was reduced in infected silver eel swimbladder tissue. In line with this observation the increase in MDA concentrations revealed an increase in oxidative stress due to the infection. However, in yellow eels the infection did not cause a decrease in the ROS defense capacity, SOD activity was even elevated in infected swimbladder, although this difference was not significant. Infections with *A. crassus* seem to compromise ROS defense only in silver eels and impair the silvering induced changes in enzyme activities and metabolite concentrations. Fazio et al. (2012) analyzed the effect of an infection with *A. crassus* on, for example, gut mass, liver mass, ocular index and the expression of several silvering related genes (including androgen receptors α and hemoglobin α-chain) in different tissues, but not including swimbladder tissue. In infected eels, 5 out of 12 silvering related parameters analyzed were affected in artificially infected eels, and the authors suggested that infected eels were in a more advanced stage in the silvering process (Fazio et al. 2012). This indicated that the infection of the swimbladder has a significant impact on the whole animal. Our data add to this picture by showing that the nematode infection prevented or at least weakened the ROS defense enhancements occurring in the swimbladder during silvering.

Noteworthy appears the observation that silvering was connected to a significant increase in SOD activity in muscle tissue, and this effect was attenuated in infected silver eel muscle. The skeletal muscle is known to produce superoxide anions at a low rate, and the rate of production is dramatically increased during activity (Reid 2001). While yellow eels typically dwell near the bottom (Tesch et al. 2003), migrating eels must continuously swim for a period of about 5–6 months, and this activity therefore can be expected to result in an increase in ROS production in muscle tissue. An increase in SOD activity may therefore be useful in order to cope with the increased oxidative stress during migration. But in contrast to swimbladder tissue this improvement in ROS defense did not involve the glutathione reaction, suggesting tissue specific differences in the strategy to cope with ROS. In infected silver eels, this increase in SOD activity was not observed demonstrating again the negative effect of the infection on eel physiology. Swim tunnel experiments revealed that eels with infected swimbladder compared with uninfected eels swim much less efficient and stop swimming much earlier than uninfected eels (Palstra et al. 2007). It could be that the reduced capacity to cope with ROS in muscle tissue in these infected eels contributed to the lower swimming performance.

In a recent study, it was shown that swimbladder tissue of freshwater fish, using the swimbladder as an air-breathing organ, also has an improved ROS defense system (Pelster et al. 2016). Accordingly, an improved ROS defense capacity appears to be a necessity for all swimbladder tissues, exposed to elevated oxygen partial pressures. Our data show that during silvering the ROS defense capacity of swimbladder tissue of the European eel is significantly improved in order to prepare for the high oxygen partial pressures encountered during the diurnal vertical migration during the spawning migration.

Our silver eels were caught in the transition zone between fresh- and seawater and therefore did not yet experience the very high hydrostatic pressure encountered in the open ocean. It, therefore, may well be that the improvement in the ROS defense capacity detected in these eels will even be enhanced when reaching the open ocean and performing the daily vertical migrations. This improvement was largely abolished by the infection of the swimbladder with *A. crassus*. In consequence, the nematode not only impairs swimming capacity of the (Palstra et al. 2007), but also the ROS defense in the swimbladder, which is essential to avoid the damaging effect of ROS during spawning migration. Our data, therefore, provide additional support for the notion that the nematode infection represents a serious threat for a successful spawning migration.

## Electronic supplementary material

Below is the link to the electronic supplementary material.

**Supplementary Figure 1** Activity of ROS-related enzymes and metabolite concentrations in muscle and swimbladder tissue of different development and infection status. (A) glutathione reductase, (B) glutathione peroxidase, (C) catalase, (D) superoxide dismutase, (E) total glutathione (GSH+GSSG). Bars represent mean values ± SD (*N* = 6). * marks *P* < 0.05 (TIFF 25515 kb)
