# Supplementary figures and images for: Anguillicola crassus impairs the silvering-related enhancements of the ROS defense capacity in swimbladder tissue of the European eel (Anguilla anguilla)
Source: J Comp Physiol B. 2016 May 4;186(7):867–77. doi: 10.1007/s00360-016-0994-0 (PMC5009179; doi:10.1007/s00360-016-0994-0)

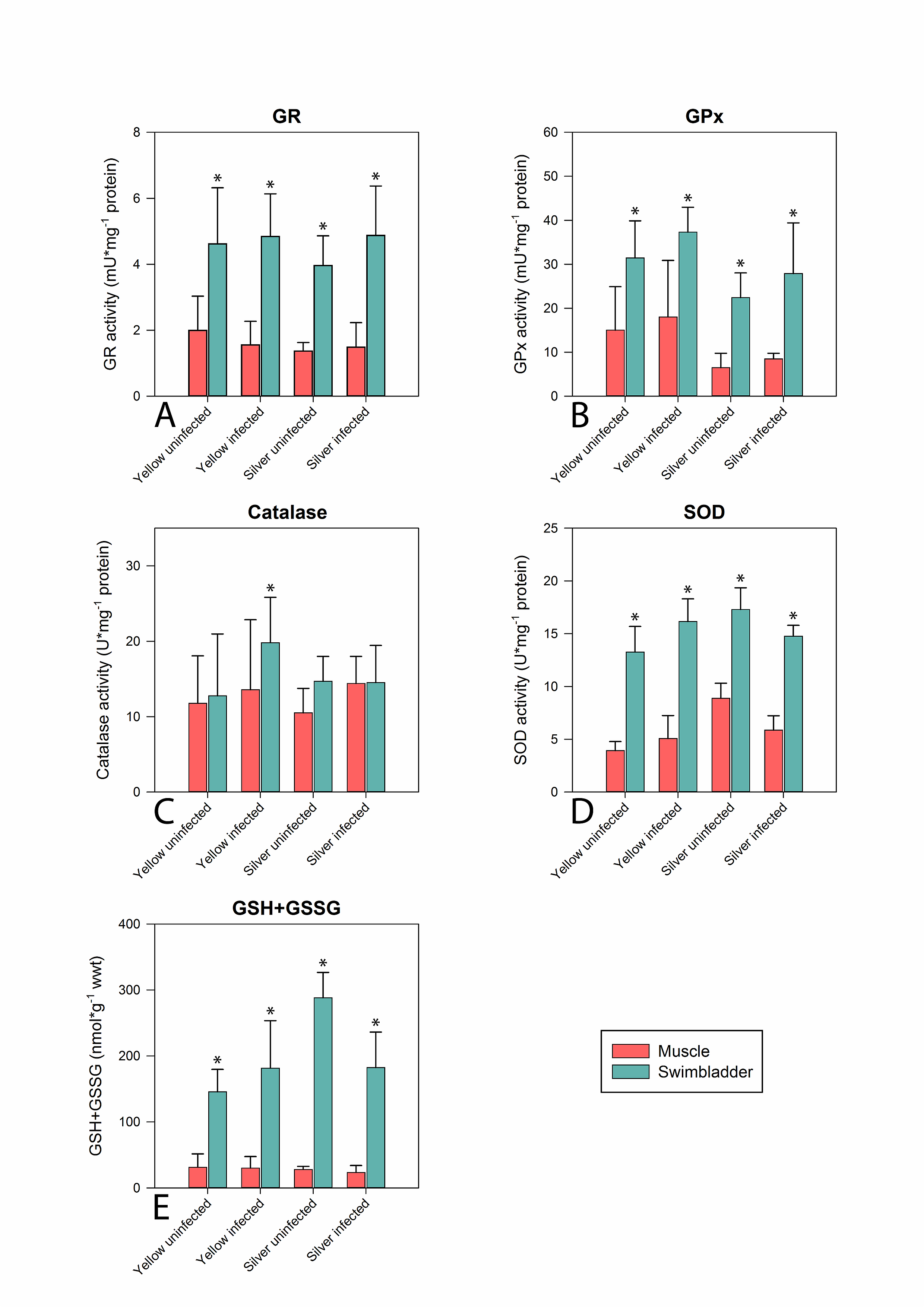

Supplement: Supplementary file 1 — Supplementary Figure 1 Activity of ROS-related enzymes and metabolite concentrations in muscle and swimbladder tissue of different development and infection status. (A) glutathione reductase, (B) glutathione peroxidase, (C) catalase, (D) superoxide dismutase, (E) total glutathione (GSH+GSSG). Bars represent mean values ± SD (N = 6). * marks P < 0.05 (TIFF 25515 kb) [file 360_2016_994_MOESM1_ESM.tif]
